# Two Validated Spectrofluorometric Methods for Determination of Gemifloxacin Mesylate in Tablets and Human Plasma

**DOI:** 10.1155/2013/137279

**Published:** 2013-05-16

**Authors:** Noha N. Atia, Ashraf M. Mahmoud, Salwa R. El-Shabouri, Wesam M. El-Koussi

**Affiliations:** ^1^Department of Pharmaceutical Analytical Chemistry, Faculty of Pharmacy, Assiut University, Assiut 71526, Egypt; ^2^Department of Pharmaceutical Chemistry, Faculty of Pharmacy, Najran University, P.O. Box 1988, Najran 61441, Saudi Arabia

## Abstract

Two new, sensitive, and selective spectrofluorometric methods were developed for the determination of gemifloxacin mesylate (GFX) in tablets and spiked human plasma. Method A was based on measurement of the enhanced fluorescence spectral behaviour of GFX in a sodium dodecyl sulphate (SDS) micellar system. In aqueous solution of acetate buffer pH 5.5, the fluorescence intensity of GFX was greatly enhanced about tenfold in the presence of SDS. The fluorescence intensity was measured at 402 nm after excitation at 274 nm. Method B was based on Hantzsch condensation reaction between the primary amino group of GFX with acetylacetone and formaldehyde in acetate buffer of pH 3.5 yielding a highly yellow fluorescent derivative. The reaction of GFX with acetylacetone-formaldehyde system solution resulted in bathochromic shift of both emission (476 nm) and excitation (420 nm) wavelengths. The fluorescence intensity was directly proportional to the concentration over the range 10–1000 ng/ml and 100–2000 ng/ml for method A and B, respectively. The proposed methods were applied successfully for determination of GFX in its tablets and spiked plasma. Therefore, these methods can be considered of real interest for reliable and practical quality control analysis of GFX.

## 1. Introduction

**Figure 1:**
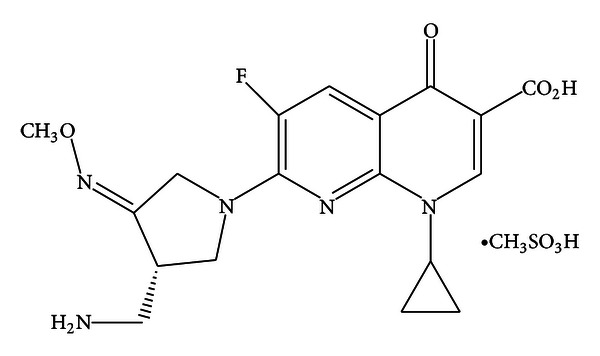
Chemical structure of gemifloxacin mesylate.

Gemifloxacin mesylate (GFX) is a fluoronaphthyridone with a novel oxime functionalised pyrrolidine (Figure 1) [1]. It possesses a dual mechanism of action by inhibiting the bacterial topoisomerase IV and gyrase enzymes, resulting in interruption of bacterial DNA synthesis. Therefore, it has broad-spectrum activity against Gram-positive and Gram-negative organisms comparable to those of its quinolone counterparts which have the same pyrrolidine side chain [1, 2]. GFX was first approved by the FDA for clinical use in 2003 for the treatment of community acquired pneumonia and acute bacterial exacerbation of chronic bronchitis [3].

Several analytical methods were reported for determination of gemifloxacin in pharmaceutical preparations or human plasma by visible spectrophotometry [4, 5], capillary electrophoresis [6], HPLC-MS-MS [7], HPLC [8], and HPTLC [9]. However, these methods showed some drawbacks such as being time-consuming, tedious, or requiring expensive instruments that limit their use in quality control and routine clinical studies in developing countries where the cost is a main concern.

Spectrofluorimetric analysis constitutes a widespread, effective technique to improve analysis selectivity and sensitivity. Although, limited literatures have been reported for the determination of gemifloxacin spectrofluorometrically [10, 11]. Micelle-enhanced spectrofluorimetric method has been reported for determination of many drugs [12–17] due to the ability of micelle formation to increase the fluorescence intensity of the weakly fluorescent compounds. Moreover, these methods introduced sensitive and nonpollutant methodology, since no organic solvents were used. Hantzsch reaction is a well-known condensation reaction that was reported as a useful pathway for determination of many drugs fluorometrically in biological fluids, air, or pharmaceutical preparations in trace amounts [18–22]. 

Therefore, the aim of this study was directed for using micelle-enhanced and Hantzsch reaction spectrofluorometric methods for developing new simple, selective, and sensitive methods for determination of GFX in its tablets and human plasma. 

## 2. Experimental

### 2.1. Instrumentation

Spectrophotometric measurements were carried out using an LS 45 luminescence spectrometer (Perkin-Elmer, UK), equipped with a 150 W Xenon lamp. Slit widths for both monochromators set at 10 nm. Data acquisition was performed by FL WinLab software, version 4.00.03 (Perkin-Elmer, UK). A 1 cm quartz cell was used. 

 A solid phase extraction (SPE) vacuum manifold with 24-position configurations (Phenomenex, USA) was used for extraction of plasma samples. Vacuum was adjusted to 5 inches for proper flow through the SPE columns. The SPE cartridge used in this study was Strata C18-U (1 cm^3^/100 mg; Phenomenex, USA).

### 2.2. Materials and Reagents

Gemifloxacin mesylate (99.8%, Tabuk pharmaceutical manufacturing Co., KSA) was used as received. The commercial formulation “Factive” (Oscient Pharmaceuticals, USA) was purchased from our local market, which was labeled to contain 320 mg of GFX per tablet. Simulated tablets were prepared in our laboratory according to similar dosage form in India “G-CIN-A” [23, 24] which are labeled to contain 320 mg of GFX and 75 mg of Ambroxol HCl per tablet. We used sodium dodecyl sulphate (SDS) (El-Nasr chemical Co., Egypt), cetrimide (Danochemo a subsidiary of Ferrosan, manufacturing chemists, Copenhagen, Denmark), beta-cyclodextrin (*β*-CD) (Winlab Laboratory chemicals reagents fine chemicals, UK). Acetylacetone was obtained from Tedia CO., USA. 34–38% Formaldehyde solution and other solvents and materials used throughout this study were of analytical grade. Double distilled water was obtained through WSC-4D water purification system (Hamilton Laboratory Milton Glass Ltd., Kent, USA) and used throughout the work. 0.1 M acetate buffer solution of pH 3.5 and 50 mM phosphate buffer pH 3 [25] were prepared and adjusted using 3505 pH-meter (Jenway, UK). Blank human plasma samples used herein were supplied from Assiut University Hospitals (Assiut, Egypt), and they were stored in deepfreezer at −80°C until analysis.

#### 2.2.1. Reagent Preparation

*SDS Solution.* 0.1 M SDS solution was prepared by dissolving 0.29 g of SDS in sufficient distilled water and diluted up to 10 mL.

*Acetylacetone-Formaldehyde Solution.* Into 5 mL calibrated flask, the reagent was freshly prepared by mixing 0.5 mL of 0.1 M acetate buffer pH 3.5, 0.5 mL acetylacetone solution, and 1 mL formaldehyde solution and completed to the mark with double distilled water. The flask was protected from light with aluminum foil.

### 2.3. Standards and Stock Solutions

#### 2.3.1. Gemifloxacin Mesylate Standard Solutions

An accurately weighed amount (25 mg) of GFX was transferred into 250 mL calibrated flask, dissolved in appropriate volume of double distilled water. Then, the void volume was completed with water to produce a stock solution of 100 *μ*g/mL. The stock solution was further diluted with water to obtain working standard solution of 5 *μ*g/mL.

#### 2.3.2. Tablets Sample Solution

Twenty tablets were weighed and finely powdered. An accurately weighed quantity of the powder equivalent to 25 mg of GFX alone or with Ambroxol in combined dosage form was transferred into a 100 mL calibrated flask and dissolved in about 40 mL of distilled water. The contents of the flask were swirled, sonicated for 5 min, and then completed to volume with water. The contents were mixed well, filtered, and rejecting the first portion of the filtrate. The prepared solutions were diluted quantitatively with water to obtain stock solution of 5 *μ*g/mL as a suitable concentration for the analysis.

#### 2.3.3. Plasma Sample Processing

*Sample Preparation.* Plasma aliquot (0.25 mL) was transferred into a 2 mL Eppendorf tubes. Subsequently, 100 *μ*L of GFX working solutions (2, 12.5, 25, 50, and 100 *μ*g/mL) was added in each tube. After gentle mixing, the void volume was completed to 1.5 mL with 50 mM Na_2_HPO_4_ (pH 3). A blank plasma sample was treated similarly.

*Solid-Phase Extraction.* A polymeric sorbent (Strata C18-U) was used to prepare the samples. Before extraction, the cartridges were prewashed with 2 × 1 mL of methanol, followed by 2 × 1 mL of distilled water. After application of the samples, the cartridges were washed with 2 × 1 ml portions of distilled water and 0.5 mL of 50 mM Na_2_HPO_4_ (pH 3). Finally, GFX was eluted with 1 mL of methanol and 50 mM Na_2_HPO_4_ (pH 3, 90 : 10 v/v). Then 0.5 mL was taken from each eluent and the general procedure was followed to obtain final concentrations of 20, 125 and 500 ng/mL for method A or 250, 500 and 1000 ng/mL for method B.

### 2.4. General Procedures

#### 2.4.1. Micelle-Enhanced Spectrofluorometric Method (Method A)

Aliquot of 10–1000 *μ*L of GFX standard solution (5 *μ*g/mL) was transferred into a series of 5 mL volumetric flasks to give final concentrations of 10–1000 ng/mL. 1 mL 0.2 M acetate buffer solution, pH 5.5, was added to each flask, followed by 100 *μ*L of 0.1 M SDS solution. The volume was completed with distilled water, the contents of the flasks were mixed well, and the fluorescence intensity was measured at 402 nm after excitation at 274 nm, against a blank solution treated similarly.

#### 2.4.2. Hantzsch Reaction Method (Method B)

Aliquot of 0.1–2.0 mL of GFX standard solution (5 *μ*g/mL) was mixed with 1 mL of acetylacetone-formaldehyde solution in a glass-stoppered tube that was protected from light with aluminum foil. The mixture was heated at 100°C for 20 min in a thermostatic water bath and after that it was cooled in an ice bath. The volume was adjusted to 5 mL with 2-propanol to provide a final concentration ranging from 100–2000 ng/mL. The fluorescence intensity was measured at 476 nm after excitation at 420 nm against a blank prepared similarly.

## 3. Results and Discussion

### 3.1. Spectral Characteristics

**Figure 2:**
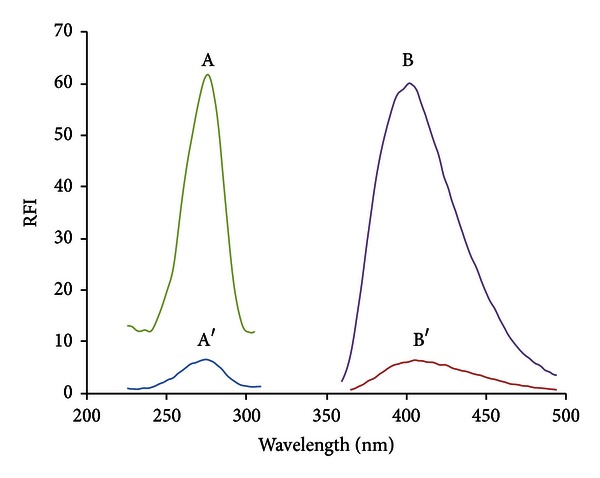
Fluorescence spectra of (A, B) GFX (500 ng/mL) in acetate buffer, pH 5.5/SDS system; (A′, B′) GFX (500 ng/mL) in acetate buffer, pH 3.5, where (A, A′) are the excitation spectra and (B, B′) are the emission spectra.

For method A, the fluorescence spectra of gemifloxacin in both aqueous and SDS systems were studied (Figure 2). GFX showed native fluorescence in aqueous solution measured at 406 nm after an excitation at 274 nm. In the presence of SDS, the fluorescence intensity of GFX was enhanced nearly tenfold in comparison with its native fluorescence intensity in aqueous medium. Moreover, the enhancement was associated with a slight blue shift (*λ*em at 402 nm). It reflects that the microenvironment around GFX is quite different from that in aqueous solution. This can be attributed to restrictions imposed on the free rotational motions which are competitive with luminescent emission [26]. 

**Figure 3:**
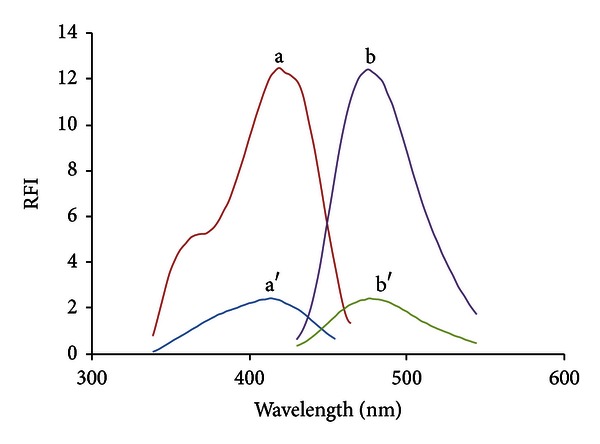
Fluorescence spectra of (a, b) gemifloxacin (1000 ng/mL) with acetylacetone-formaldehyde reagent solution, (a′, b′) acetylacetone-formaldehyde reagent, where (a, a′) are the excitation spectra and (b, b′) are the emission spectra. *λ*ex/*λ*em = 420/476 nm.

For method B, GFX reacts with acetylacetone and formaldehyde in an acidic-buffered medium yielding a highly yellow fluorescent product. The fluorescence intensity of the product was measured at 476 nm after excitation at 420 nm (Figure 3). The notable advantage of this reaction is the enhanced red shift in the excitation and emission wavelengths that improves the selectivity of GFX.

### 3.2. Optimization of Experimental Conditions

All different experimental factors influencing the development of the fluorescent product were carefully studied and optimized. Such factors were changed individually while others were kept constant. These factors were pH, volume of the reagent, temperature and diluting solvent.

#### 3.2.1. Micelle-Enhanced Spectrofluorometric Method

**Figure 4:**
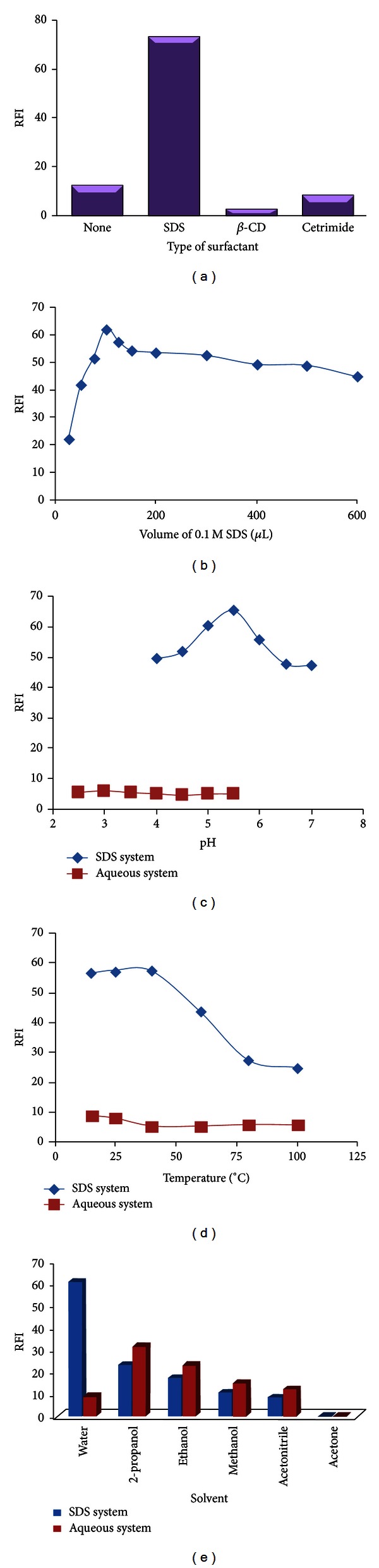
Effect of the different optimization factors. (a) Type of surfactant, (b) concentration of SDS surfactant, (c) pH, (d) temperature, and (e) diluting solvents on RFI using micelle-enhanced spectrofluorometric method.

The fluorescence properties of gemifloxacin in various micellar media were studied using anionic (SDS), cationic (cetrimide) and nonionic (*β*-CD) surfactants. It was observed that the fluorescence intensity of GFX showed decrease or no significant effect by using non-ionic or cationic surfactants. On the other hand, there was an obvious enhancement of the fluorescence intensity of GFX in the presence of SDS about tenfold in comparing with its aqueous solution (Figure 4(a)). Therefore, the influence of SDS on the RFI was studied using increasing volumes of 0.1 M SDS. It was found that increasing volumes of SDS solution resulted in a corresponding increase in RFI up to 100 *μ*L, after which gradual decrease in RFI was attained. Therefore, 100 *μ*L of 0.1 M SDS solution was selected as the optimum volume (Figure 4(b)). It was noted that, the SDS aggregation equilibrium in presence of GFX in our optimal experimental conditions showed a quite different behavior than pure SDS aqueous solution. For GFX-SDS system, the fluorescent response showed maximum intensity at 2  ×  10^−3^ M which is lower than the reported critical micellar concentration (cmc) of SDS (8 × 10^−3^ M) [27]. The change in the cmc of SDS in our results suggests the formation of mixed aggregates at concentrations below the reported cmc that was in concordance to other authors whose reported similar behavior for SDS systems [28–30].

The influence of pH on the micelle-enhanced fluorescence of GFX was studied carefully. The fluorescence intensity was maximal in the pH interval of 5.0–6.0. Thus, pH 5.5 was selected to be the most successful for further studies (Figure 4(c)). It may be suggested that at this pH value (5.5) GFX is present in its protonated form, because the fluorescence intensity of protonated species is always higher than that of neutral species [31, 32]. This can be inferred that protonated forms interact more strongly with the anionic micelles of SDS than the neutral forms of the drugs. Different buffer solutions (acetate, phosphate, and Torell and Stenhagen) were tested. The results revealed that the acetate buffer solution of pH 5.5 achieved the maximum fluorescence intensity, and the variation in the buffer concentration did not show any significant change in the fluorescence intensity. A 0.2 M acetate buffer was selected to obtain an adequate buffering capacity for further measurements.

The ionic strength can also influence significantly the solubilization of a drug in micellar solutions, especially in case of ionic surfactants [33]. Therefore, the effect of the addition of inert salt such as KCl on micellar solutions of GFX was tested. It was found that an increase of concentration above 5 × 10^−2^ M provoked a clouding phenomenon to the system, and below this value, no significant effect was observed.

Another factor that affects the fluorescence intensity of the micellar system of GFX is the temperature. The effect of temperature was studied in the range 25–100°C in a thermostatically controlled water bath. It was found that increasing the temperature resulted in a decrease in the RFI (Figure 4(d)). This effect can be explained by higher internal conversion as the temperature increases, facilitating nonradiative deactivation of the excited singlet state [34]. The results indicated that the fluorescence intensity was immediately developed at room temperature and remained stable for at least 2 hr.

Finally, the influence of different diluting solvents (water, methanol, ethanol, isopropanol, acetonitrile, or acetone) on the fluorescence intensity of GFX-SDS system was also investigated (Figure 4(e)). The results revealed that water was the best solvent for dilution in presence of SDS, as it gave the highest RFI and the lowest blank reading, while distinct and sharp decrease in the relative fluorescence intensities was observed in the SDS system using other solvents. This effect is attributed to their denaturating effect on the micelles, where short-chain alcohols (methanol, ethanol, and propanol) are solubilized mainly in the aqueous phase and affect the micellization process by modifying the solvent properties. Addition of these alcohols also results in a reduction of the size of the micelles but with a progressive breakdown of the surfactant aggregate at very high concentration [35].

#### 3.2.2. Hantzsch Reaction Method

**Figure 5:**
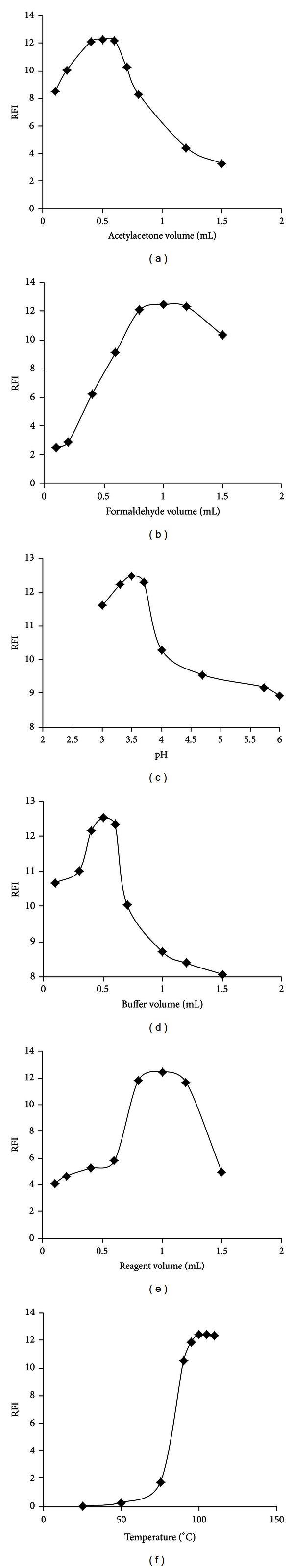
Effect of the different optimization factors. (a) Volume of acetylacetone, (b) volume of formaldehyde, (c) pH, (d) volume of buffer, (e) volume of reagent components, and (f) temperature on RFI using Hantzsch reaction.

Effect of reagents composition; the effect of acetylacetone and formaldehyde reagents volume in the final reagent solution were studied individually to show their influence on RFI (Figure 5). Different volumes ranging from 0.1 to 1.5 mL of either acetylacetone or formaldehyde solution (34–38%) were tested. A reagent composed of both 0.5 mL of acetylacetone and 1 mL of formaldehyde resulted in the maximum RFI and it was selected for further studies (Figures 5(a) and 5(b)).

In order to select the most appropriate pH, the reaction was carried out at different pHs. First, the pH of the reaction medium was changed over pH range 3–6 using 0.1 M acetate buffer to obtain the highest RFI of the resulted product. The maximum RFI was obtained at pH 3.5 (Figure 5(c)). Then, a series of different buffer systems (acetate, Mcllvaine, and Torell and Stenhagen buffers) of pH 3.5 was studied; the results indicated that acetate buffer was still the superior one. Also different volumes of the optimum buffer solution ranging from 0.1 to 2 mL were tested to obtain the maximum sensitivity. 0.5 mL of acetate buffer was selected for further investigations (Figure 5(d)). 

After individual optimization of the reagent components, the effect of the final volume of the reagent system solution was studied over the range 0.1–1.5 mL. It was found that 1 mL of the reagent system was the optimum volume for further studies (Figure 5(e)). 

The optimum temperature for the reaction was determined by investigating the RFI at different temperatures in the range 25–110°C in a thermostatically controlled water bath. The results revealed that the heating step is essential. The maximum RFI was reached after 20 min at 100°C (Figure 5(f)). Moreover, to select the most appropriate diluting solvent, the reaction mixture was diluted using different solvents (water, methanol, ethanol, 2-propanol, acetonitrile, or acetone). The results showed that 2-propanol was the best solvent for dilution as it achieves the highest RFI.

### 3.3. Method Validation

The method was validated according to ICH guidelines of the validation of analytical methods [36]. All results were expressed as percentages, with *n* representing the number of values. Microsoft office excel 2007 was used for statistical analysis. A 5% significance level was used for evaluation.

#### 3.3.1. Linearity, Limits of Detection, and Quantitation

Under the optimum conditions, linear plots with good correlation coefficients (0.9999 and 0.9988) were obtained in the concentration ranges of 10–1000 and 100–2000 ng/mL for micelle-enhanced fluorescence and Hantzsch reaction methods, respectively. The limits of detection (LOD) and quantitation (LOQ) were determined using the formula: LOD or LOQ = *κ*SD_*a*_/*b*, where *κ* = 3.3 for LOD and 10 for LOQ, SD_*a*_ is the standard deviation of the intercept, and *b* is the slope. The LOD values were 2.32 and 20.45 ng/mL for micelle-enhanced fluorescence and Hantzsch reaction methods, respectively. The parameters for the analytical performance of the proposed method are summarized in Table 1. 

#### 3.3.2. Precision and Accuracy

The precision of the proposed methods was determined by replicate analysis of six separate sample solutions at three concentration levels of GFX. The relative standard deviations (RSD) were 0.82–1.86 and 0.44–1.90% for micelle-enhanced fluorescence and Hantzsch reaction methods, respectively.  Table 2 indicates the good reproducibility of the proposed methods. The accuracy of both methods was determined by investigating the recovery of GFX at three concentrations levels covering the specified range (six replicates of each concentration). The results shown in Table 2 depict good accuracy for the proposed methods.

#### 3.3.3. Robustness

It was estimated by testing the susceptibility of measurements to deliberate variation of the analytical conditions. It was found that minor changes that may take place during the experimental operation did not affect the RFI of both methods. The results for the proposed methods are summarized in Table 3.

#### 3.3.4. Specificity

The specificity of the proposed methods was investigated by considering the interference liabilities from Ambroxol HCl in the combined dosage form. Results present in Table 4 indicate that no interference from Ambroxol HCl was observed.

### 3.4. Applications of the Proposed Methods

#### 3.4.1. Determination of GFX in Dosage Forms

The applicability of the proposed methods was tested by the determination of GFX in its marketed product (Factive Tablet) and simulated tablet with ambroxol HCl as a combined dosage form. The results obtained are accurate and precise as indicated by the excellent percentage recovery (Table 4).

Statistical analysis of the results obtained by the proposed methods and those given by reference method [9] was performed using the Student's *t*-test and the variance ratio *F*-test. The calculated values did not exceed the theoretical ones, indicating no significant difference between the compared methods regarding accuracy and precision, respectively. The recovery results of simulated tablets also indicated the selectivity of the proposed methods for GFX in presence of Ambroxol HCl (Table 4). Therefore, the proposed methods are recommended for the quality control analysis of GFX in its pharmaceutical preparations.

#### 3.4.2. Determination of GFX in Plasma

The high sensitivity of the proposed methods allowed the determination of GFX in spiked human plasma. Allen et al. [37] studied the pharmacokinetic parameters of GFX in healthy volunteers after a single oral dose administration. This method indicated that the maximum concentration (*C*_max⁡_) of the drug was achieved approximately one hour after dosing, and the mean *C*_max⁡_ value was found as 1.48 ± 0.39 *μ*g/mL following a single oral dose of 320 mg GFX. Therefore, the drug level in plasma is within the working linearity range of the proposed method (Table 1).

Recently, solid phase extraction (SPE) becomes the most commonly used technique for sample extraction (especially of biological origins) due to its environmental safety. Compared to liquid-liquid extraction [11], the SPE method has several advantages such as making complete phase separations, high quantitative recoveries, no need for using expensive breakable specialty glassware, and disposal of large quantities of organic solvents. Hence, SPE represents efficient separation of interfering substances from analytes without tedious and time-consuming steps. Therefore, a polymeric SPE cartridge was used to prepare the samples in this study. 

Hobara et al. introduced simple SPE method for determination of fleroxacin in rat plasma [38]. This method was used with slight modification to be more compatible with our method. This modification includes increasing the volume of spiked plasma to ensure the efficiency of this method (as the reported method was proposed for real plasma samples). This simple and efficient extraction procedure introduces successful method for GFX analysis in human plasma.  Table 5 indicates that the obtained results are satisfactorily accurate and precise.

## 4. Suggested Mechanism of the Proposed Methods

### 4.1. Micelle-Enhanced Spectrofluorometric Method

**Scheme 1:**
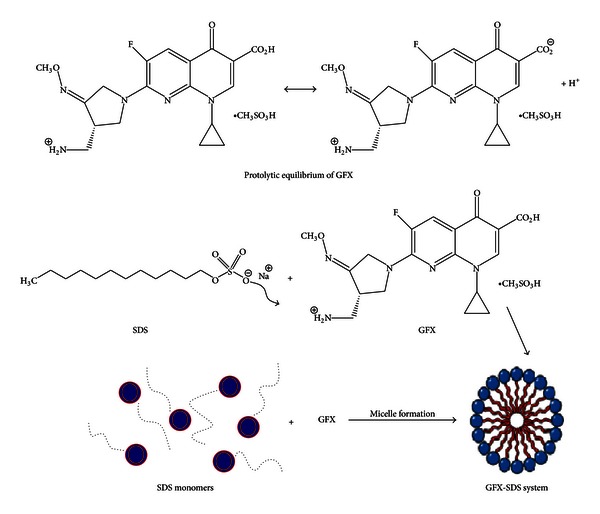
The suggested mechanism for GFX-SDS micelle formation.

This method has been reported for determination of many fluoroquinolones [12–15] and other compounds [16, 17] using different types of surfactants. In the presence of SDS anionic micelles, gemifloxacin is solubilized in a more favourable microenvironment that produces an important improvement in fluorescence intensity. GFX possesses two ionizable functional groups: a carboxylic group (pKa_1_-6.5) and a basic amino group (pKa_2_-8.9). It is thought that in the micellar phase, SDS could form an ion paired species with GFX through the interaction between the SDS negative sulphonyl (OSO_3_^−^) group and the protonated basic amino group of GFX (Scheme 1).

### 4.2. Hantzsch Reaction Mechanism

**Scheme 2:**
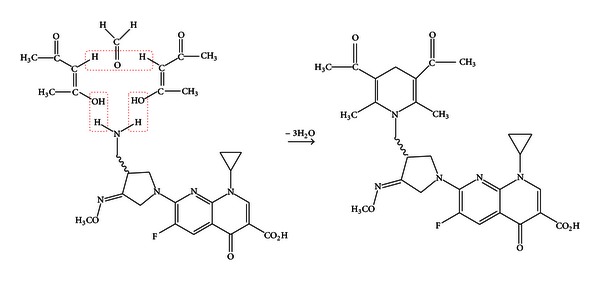
The suggested mechanism for the Hantzsch reaction between gemifloxacin and acetylacetone-formaldehyde reagent.

Hantzsch reaction occurs between acetylacetone (*β*-diketone derivative) in combination with formaldehyde and the aliphatic amino group containing compounds [18–22]. GFX reacts via its primary aliphatic amino group toward this combination in an acidic-buffered medium yielding a highly yellow fluorescent condensation product (Scheme 2). 

## 5. Conclusion

The proposed methods are quite simple, accurate, precise, and do not require tedious extraction procedure. Considering the limits of detection and/or concentrations ranges, the developed methods are highly sensitive. Moreover, the micelle-enhanced spectrofluorometric method has additional advantages that it is rapid and nonpollutant (organic solvents free) that can be used as an alternative to the more time-consuming, expensive HPLC methods. The proposed methods were successfully applied for determining GFX in its tablets or plasma samples without any interference from the matrices. Thus, it can be effectively used for routine analysis of GFX in pharmaceutical industries, hospitals, and research laboratories. 

## Figures and Tables

**Table 1:** Analytical parameters for the determination of GFX using the two proposed methods.

Parameter	Micelle-enhanced spectrofluorometric method	Hantzsch reaction method
Range (ng/mL)	10–1000	100–2000
Intercept (*a*) ±SD^a^	−0.50 ± 0.10	1.28 ± 0.07
Slope (*b*) ± SD^a^	0.13 ± 2 × 10^−4^	0.01 ± 4 × 10^−3^
Correlation coefficient (r)	0.9999	0.9988
LOD (ng/mL)	2.32	20.45
LOQ (ng/mL)	7.74	68.18

^a^Mean of six determinations.

**Table 2:** Intraday and interday precision of the proposed methods.

Method	Conc. (ng/mL)	Recovery (%) ± SD^a^	Intraday precision		Interday precision	
			Mean ± SD^a^	%RSD	Mean ± SD^a^	%RSD
	20	99.86 ± 1.17	100.63 ± 1.13	1.18	99.10 ± 1.50	1.51
Micelle-enhanced spectrofluorometric	125	96.49 ± 0.40	99.82 ± 0.82	0.82	99.63 ± 1.85	1.86
	500	99.76 ± 1.66	100.34 ± 1.09	1.08	100.23 ± 1.43	1.42

	250	99.24 ± 2.25	99.31 ± 0.58	0.58	98.36 ± 1.89	1.90
Hantzsch reaction	500	100.71 ± 1.98	100.56 ± 0.70	0.70	100.71 ± 1.44	1.43
	1000	99.35 ± 1.44	99.86 ± 0.44	0.44	99.57 ± 0.82	0.82

^a^Mean of six determinations.

**Table 3:** Robustness of the proposed methods.

Method	Experimental parameter variation	Recovery (%) ± SD^a^
	No variation^b^	101.07 ± 0.34
	SDS volume (*µ*L)	
	90	96.10 ± 1.13
	110	99.16 ± 0.44
	pH	
Micelle-enhanced spectrofluorometric	5.3	101.23 ± 1.27
	5.7	98.52 ± 0.55
	Temperature (°C)	
	20	102.13 ± 0.91
	30	100.81 ± 1.92

	No variation^b^	101.57 ± 0.53
	pH	
	3.3	100.05 ± 1.83
	3.7	101.02 ± 2.79
	Acetylacetone volume (mL)	
	0.4	99.16 ± 1.40
	0.6	99.86 ± 0.91
Hantzsch reaction	Formaldehyde volume (mL)	
	0.8	97.60 ± 0.92
	1.2	100.90 ± 2.30
	Temperature (°C)	
	95	97.06 ± 1.40
	105	102.36 ± 1.90
	Heating time (min)	
	18	99.31 ± 2.74
	22	102.33 ± 1.83

^a^Mean of three determinations.

^b^Following the general assay procedure conditions.

**Table 4:** Determination of GFX in its pharmaceutical dosage forms using the proposed methods.

Dosage form	Recovery % ± SD (*n* = 4)		
	Micelle-enhanced spectrofluorometric method	Hantzsch reaction method	Reported method^a^
	99.78 ± 0.88	99.44 ± 0.81	100.09 ± 0.50
Factive	*t* = 0.61^b^	*t* = 1.35^b^	
	*F* = 3.17^b^	*F* = 2.69^b^	

	99.07 ± 0.66	100.85 ± 0.56	100.14 ± 1.05
Simulated tablets contain Ambroxol HCl	*t* = 1.72^b^	*t* = 1.19^b^	
	*F* = 2.49^b^	*F* = 3.49^b^	

^a^Reference [9].

^b^Theoretical value for *t* and *F* at 95% confidence limit, *t* = 2.45 and *F* = 9.23.

**Table 5:** Determination of GFX in spiked human plasma samples using the proposed methods.

Method	Spiked amount (ng/mL)	Found (ng/mL)	Recovery % ± SD^a^
	20	19.52	97.60 ± 0.92
Micelle-enhanced spectrofluorometric	125	123.98	99.18 ± 1.23
	500	495.50	99.10 ± 0.69

	250	248.11	99.25 ± 0.97
Hantzsch reaction	500	498.25	99.65 ± 1.42
	1000	1007.07	100.71 ± 0.88

^a^Mean of five determinations.
